# Sex differences in cervical disc height and neck muscle activation during manipulation of external load from helmets

**DOI:** 10.1113/EP091996

**Published:** 2024-08-09

**Authors:** Nai‐Hao Yin, Irene Di Giulio, Peter D. Hodkinson, Federico Formenti, Ross D. Pollock

**Affiliations:** ^1^ Centre for Human and Applied Physiological Sciences King's College London London UK; ^2^ Nuffield Department of Clinical Neurosciences University of Oxford Oxford UK; ^3^ Department of Biomechanics University of Nebraska Omaha Omaha, NB USA

**Keywords:** EMG, fatigue, helmet, neck biomechanics, ultrasound

## Abstract

Neck pain associated with helmet‐wear is an occupational health problem often observed in helicopter pilots and aircrew. Whether aircrew helmet wearing is associated with physiological and biomechanical differences between sexes is currently unknown. This study investigated neuromuscular activation patterns during different helmet‐wearing conditions. The helmet load was manipulated through a novel Helmet Balancing System (HBS) in healthy, non‐pilot male and female participants (*n* = 10 each, age 19–45 years) in two phases. Phase A assessed the acute effects of helmet‐wear on neck muscles activation during head movements. Phase B examined changes in muscle activity and cervical disc height after wearing a helmet for 45 min. In Phase A, muscle activity was similar between sexes in many movements, but it was higher in female participants when wearing a helmet than in males. The HBS reduced muscle activity in both sexes. In Phase B, female participants exhibited a greater level of muscular fatigue, and male participants’ cervical disc height was significantly decreased [5.7 (1.4) vs. 4.4 (1.5) mm, *P* < 0.001] after continuous wearing. Both sexes showed no significant change in muscle fatigue and disc height [male: 5.0 (1.3) vs. 5.2 (1.4) mm, *P* = 0.604] after applying HBS. These findings demonstrate sex‐specific physiological and biomechanical responses to wearing a helmet. They may indicate different postural and motor control strategies, associated with different neck pain aetiologies in male and female aircrew, the knowledge of which is important to reduce or prevent musculoskeletal injuries associated with helmet wearing.

## Abstract (highlights)

- **What is the central question of this study?**
  Do sex differences exist in the neck physiological response to helmet‐wearing?
- **What is the main finding and its importance?**
  Sex differences exist in both the acute response and after 45 min of helmet wearing: during a given head movement, female participants’ muscle activity was greater than male participants’ and females also demonstrated greater muscular fatigue after continuous helmet‐wear than males while cervical disc height showed a significant reduction after 45 min helmet‐wear in males only. These findings could provide insight into future training or injury prevention strategy for pilots.

## 1 INTRODUCTION

While males and females have similar anatomical neck structures, their responses to head/neck loading may vary due to sex‐specific geometrical and morphological differences. In general, males have thicker necks and heavier heads (Covassin et al., 2016; Marar et al., 2012; Walton et al., 2009), and have higher average neck muscle strength than females (Chiu et al., 2002; Versteegh et al., 2015). In males and females matched for seated height and head circumference, vertebral body width and the distance between the anterior and posterior fourth cervical vertebral border of the facet joint are significantly greater in males (Stemper et al., 2008). Neck muscle strength differences exist between males and females matched for body height and neck length (Vasavada et al., 2008), suggesting that head movements might be mechanically more demanding for females than males under the same external load. Furthermore, compared to males, females generally have higher baseline sternocleidomastoid (SCM) electromyography (EMG) amplitude and higher level of EMG in anticipation of a perturbation in neck extension movement (Alsalaheen et al., 2019). Some of these differences may, therefore, be associated with females being more likely to suffer from whiplash injury after a rear collision motor vehicle incident (Walton et al., 2009), having a higher incidence of concussion in sports (Covassin et al., 2016; Marar et al., 2012), and being more likely to report neck pain and discomfort during work (Nordander et al., 2016). These morphological, mechanical and motor control differences between sexes could potentially explain the higher incidence rate of neck pain in female than male individuals (for review see Côté, 2011; GBD 2021 Neck Pain Collaborators, 2024).

Helmets are crucial safety equipment for pilots, providing vital head and face protection and impact absorption capacity. By providing a base to mount equipment on, the helmet also plays a vital role in communication, vision and life support. However, these advantages come at the price of the increased weight (∼3 kg, with night‐vision goggles and counterweight attached), and impose a large burden on the neck musculoskeletal tissues, predisposing pilots to neck pain. Neck pain prevalence among military helicopter pilots can range from 20% to 80% (Äng & Harms‐Ringdahl, 2006; Harrison et al., 2011; van den Oord et al., 2010), much higher percentages compared with the general population globally (age‐standardised rate between 2000 (males) and 2890 (females) per 100,000; GBD 2021 Neck Pain Collaborators, 2024). It is generally agreed that both long flight hours and increased helmet weight are associated with neck pain, with little correlation between body dimension or neck strength and neck pain (Harrison et al., 2012; Vail et al., 2021; Walters et al., 2012). In addition, there is a high incidence (19.0–41.5%) of cervical spine pathology among pilots (Aydog et al., 2004; Byeon et al., 2013; Landau et al., 2006). Through biomechanical and epidemiology studies of labourers with habitual head load‐carrying, it is known that long‐term axial head‐loading leads to cervical degenerative changes (88.6% vs. 22.9%; Jager et al., 1997) and greater age‐related disc height decline (Joosab et al., 1994), suggesting that axial loading could accelerate or exacerbate age‐related spinal degeneration. In this context, strategies and devices to unload the helmet weight are being developed, such as the Helmet Balancing System (HBS) utilising pneumatic pressure and an external attachment on the helmets to exert lifting force, aimed at reducing the risk of neck pain in pilots. However, given the disproportionally male dominance in both the military and aviation fields, there is a lack of study of female participants; for example, only 3 of 88 (Walters et al., 2012) and 5 of 40 (Harrison et al., 2011) participants were females in studies of neck pain in aircrew. Measuring helmet‐related physiological responses on neck musculoskeletal tissues among healthy non‐pilot females could be a first step towards understanding this problem.

This study aims to investigate whether there are sex differences in muscle activation patterns and spinal disc height when changing the load imposed on the head through a helmet, and whether applying a prototype unloading device can reduce or mitigate these changes among healthy non‐pilot participants of both sexes. Our first hypothesis was that males and females demonstrate different neck muscle activation patterns and changes in cervical disc height in response to different head/helmet load under acute dynamic and static conditions for 45 min. Our second hypothesis was that unloading the helmet weight through an external suspension arm (prototype developed for use by helicopter pilots) reduces the impact of standard helmet‐wearing conditions.

## 2 METHODS

### 2.1 Participants

This study was approved by King's College London research ethics committee (HR/DP‐22/23‐34182) and advertised through internal circulars. Exclusion criteria were age below 18 or over 60 years; history of head/neck, upper extremity, upper and lower back musculoskeletal injuries or major operations; and having consistent, chronic neck pain or headache for the past 6 months. Participants provided written informed consent, and testing was conducted in accordance with the principles of the *Declaration of Helsinki* (World Medical Association, 2013).

### 2.2 Procedures

**FIGURE 1:**
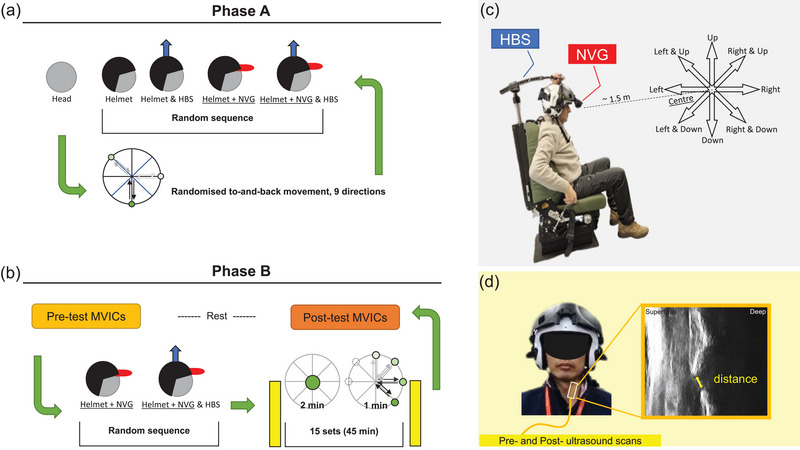
Study protocol. (a) Schematic illustration of Phase A experimental protocol. (b) Schematic illustration of Phase B experimental protocol. (c) The representative seat, Helmet Balancing System (HBS), the simulated night‐vision goggles (NVG) and the schematic drawing of the nine environmental targets. (d) The region where ultrasound images were taken and the representative ultrasound image of measuring the anterior C5–C6 intervertebral disc height in the sagittal plane.

The study consisted of two phases and participants were required to visit the laboratory twice, with a minimum of 1 week interval. In the first visit, the participants were fitted with a helmet (size: medium, medium‐long or large; mean weight 1.75 kg with maximal weight difference between sizes <50 g; Gentex Alpha 900, Key Survival Equipment, Derby, UK) by an individual trained in helmet fitting and given time to become accustomed to the helmet. Bilateral surface EMG was recorded from the SCM, anterior scalene (Sca), upper trapezius (UT) and splenius capitis (SpC) muscles (Trigno Wireless Biofeedback System and Trigno Quattro Sensor; Delsys Inc., Natick, MA, US). The electrode locations were placed according to the previous reports, also to support the comparison/contrast of results. For SCM and Sca muscles, the electrodes were placed on the muscle bellies in relation to the reference line drawn between the mastoid process and the sternal notch (Falla et al., 2003). Electrodes were placed on the muscle bellies at the midpoint between acromion and C7 for UT (Hermens et al., 1999), and the posterior neck below hair line for SpC muscle (Camp et al., 2017). Prior to electrode placement, the skin was lightly abraded and cleaned. Following this, maximum voluntary isometric contractions (MVICs) against manual resistance were performed in five directions (forward flexion, right side‐bending, left side‐bending, shoulder elevation and upper neck extension) while seated upright. The participants were asked to keep their arms at the side and pushed their heads against manual resistance provided by the researcher for at least 5 s. The other hand of the researcher was placed on the far side of the participant's shoulder for stabilising the trunk if needed. In total three contractions were performed in each direction with at least 1 min rest between each contraction. The participant was then seated with sitting height adjusted so that their eye level was horizontal to the Centre target (see below, Figure 1c). To replicate the head movements that may be performed by helicopter pilots, nine visual targets were fixed to the wall or floor around the seat approximately 1.5 m away and encompassed the range of motion pilots could experience during flight (Figure 1c), with the target set‐up being similar to that previously reported (Dibblee et al., 2015; Harrison et al., 2016).

Phase A (Figure 1a) investigated the acute effect of helmet‐wear on the neuromuscular characteristics of the neck muscles. In total, five conditions were tested: (a) no helmet (the first, control condition tested), (b) helmet only, and (c) helmet with night vision goggles (NVG) attached, representing the greatest total mass that aircrew could experience. Conditions (b) and (c) were performed with and without the use of HBS, a prototype pneumatic device that exerts lifting force to reduce the helmet weight imposed on the neck (detailed explanation below, paragraph ‘Helmets and Helmet Balancing System’). The order of the helmet conditions tested was randomised. Each condition involved nine head movements, with the centre direction being the first while the other eight movements were performed in a random order. Each head movement started in the central position with the participants instructed to smoothly, at a self‐selected speed, move and align their head with the selected visual target, hold for ∼3 s and then smoothly return to the central position. An inertial measurement unit (Trigno Duo Sensor, Delsys) was placed at the front of the helmet just above the attachment site of the NVG (or on the forehead, during no helmet condition) to determine the range of motion of the movement. Phase A ended when all five head/helmet conditions were tested.

Phase B (Figure 1b) investigated the changes in muscle activity and spinal structures after continuously wearing a helmet for 45 min. During this phase, two conditions were tested: (a) helmet with NVG, and (b) helmet with NVG and HBS. The order of the conditions was randomly determined. Immediately after putting on the helmet, an ultrasound probe was placed vertically at the left anterior neck region (along the region medial to the clavicle head of SCM muscle, lateral to the trachea and inferior to the thyroid) to scan the intervertebral disc height (Zheng et al., 2014) for approximately 1 min with multiple (at least three high‐quality) images saved. Probe position was marked using a marker pen to ensure repeatability. At the beginning of the 45 min period, the participant was instructed to look straight ahead at the central position for 1 min. After 2 min, the participant was instructed to turn their head to align with each of the nine targets in order (moving from the central position to look at each target for ∼3 s, then back to the central position and then immediately look at the next target). This sequence was repeated every 3 min, so the head movements were performed 15 times. Post‐test ultrasound scans were taken immediately after the last set of head movements. After helmet removal, another set of MVICs was performed. The participant was given at least an hour's rest to ensure no carryover fatigue before starting the next test condition.

### 2.3 Helmets and Helmet Balancing System

In the NVG conditions, the weight and mass distribution of NVG were simulated by placing a representative mass on the NVG mount of the helmet and having a counterweight attached to the rear of the helmet; with both elements attached, a total mass of 1.1 kg was added. To connect with the HBS, a carbon fibre handle was attached externally to the top of the helmet, with adjustable hinges at both sides, just slightly in front of the ear region. The HBS was fixed to the back of the seat and had a vertical pneumatic column the top of which attached to a three‐tier carbon fibre arm that could extend up to ∼2 m forward and fixed to the top of the carbon fibre handle on the helmet. A manually operated pump was placed at the right side of the seat for adjusting the pneumatic pressure and hence the lifting force (Figure 1c). Based on the guidelines of the manufacturer (Buizza Mazzei Agency, S.r.l, Rome, Italy), the pressure for each trial was set at 1.4 and 1.9 bar for the helmet‐only and helmet plus NVG conditions, respectively – based on the calculation of the internal dimension of the pneumatic column that is equivalent to the mass of the helmet only (1.75 kg) and helmet with NVG attachment (2.85 kg).

### 2.4 EMG data collection and analysis

Raw EMG signals were recorded using the system default setting at 2222 Hz, then processed by applying a fourth‐order Butterworth band‐pass filter with cut‐off frequencies of 20 and 450 Hz (di Giulio et al., 2013; Jakobsen et al., 2012). The root mean square (RMS) was calculated for each epoch (500 ms) of the data. The RMS values were then normalised to the pre‐test MVICs of the respective muscles. For Phase A, the normalised RMS EMG from the four helmet‐wearing conditions were further normalised to the head‐only condition. We only measured the range of motion and reported the RMS EMG data when the head moved from the centre to the target position. The beginning of the movement was defined by observing an averaged acceleration over twice the standard deviation of the previous 0.1 s window in the main movement axis. The end of the condition was defined similarly, but with a negative acceleration. Range of motion was then calculated, with drift corrected using an in‐house code, and reported in the three axes. The duration for averaging the RMS EMG was determined by the derived range of motion of the main movement axis. For Phase B, RMS EMG and median frequencies were calculated and averaged from the 3 s plateau during the MVICs. Median frequency was obtained by applying a fast Fourier transform (FFT) to the filtered EMG data during the MVICs to obtain the power spectral density of the EMG signal. Median frequency was defined as the frequency at which half of the total power lies. All the EMG and accelerometery data analyses were performed using an in‐house MATLAB (version 2022a. MathWorks, Natick, MA, USA) script with the build‐in Signal Processing Toolbox and Sensor Fusion and Tracking Toolbox.

### 2.5 Ultrasound data collection and analysis

All the scans were performed with the helmet on (with or without NVG) and participants looking forward (Centre). A portable B‐mode ultrasound (MicrUs and Echo Wave II software; TELEMED Medical Systems, Milan, Italy) with a 12 MHz linear array transducer (LD12‐5L40S‐3, measurement depth 40 mm) was used to measure cervical disc height at the C5–C6 level. The probe was placed in a configuration similar to that presented in previous reports (Zheng et al., 2016; Zheng et al., 2014). All images were acquired and stored for offline analysis using an in‐house MATLAB (version 2022a) script with the build‐in Image Processing Toolbox. Images were automatically contrasted and both the most distal point of C5 and the most proximal point of C6 were manually selected, and the pixel distance between the two points was calculated and converted to distance (in millimetre). Calculated straight‐line distance from the three images with the best quality during the scan were averaged. There were four trials (F:M = 2:2), which we could not obtain acceptable ultrasound images due to technical issues, and as the result, only eight participants for each sex were included in the final analysis.

### 2.6 Statistical analysis

All statistical analysis was performed using SPSS Statistics (version 28, IBM Corp., Armonk, NY, USA). An independent Student's *t*‐test was conducted to compare male and female baseline characteristics, RMS EMG and range of motion of the no‐helmet condition in Phase A, and pre–post changes of the spinal disc height in Phase B. ANOVA with Tukey's test for *post hoc* analysis was used for range of motion, RMS EMG of the four helmet conditions (Phase A), post‐test RMS EMG and post‐test median frequency (Phase B) to test the main effect from sexes and from conditions with or without HBS. Pearson's correlation coefficient was calculated to measure the association between biomechanical variables and participants’ body height and weight. Data are presented as mean and standard deviation. The significance level was set at 0.05.

## 3 RESULTS

A total of 10 males (mean age: 26.8 years, range: 19–45 years) and 10 females (mean age: 26.1 years, range: 19–39 years; *P* = 0.843) participated in the study. Males were taller [1.79 (0.06) vs. 1.65 (0.07) m, *P* = 0.003] and heavier [76.1 (12.4) vs. 62.0 (9.0) kg, *P* = 0.021] than females.

### 3.1 Phase a range of motion

During the no helmet condition, females demonstrated smaller range of motion when looking Right [64.9 (9.4) vs. 74.3 (7.2) degrees (deg), *P* = 0.024], Right and down [rotation: 71.2 (10.0) vs. 81.7 (10.3) deg, *P* = 0.032; flexion: 24.1 (7.8) vs. 34.4 (7.1) deg, *P* = 0.006] and Left and down [flexion: 32.0 (5.6) vs. 37.8 (8.3) deg, *P* = 0.002] than males. For all the helmet conditions, females showed a reduced range of motion than males except when looking Up and left (Table 1 and Supplementary Table S1). Applying the HBS further reduced the range of motion in both sexes with or without NVG‐attached conditions (Tables 1 and Supplementary Table S1).

**TABLE 1:** Range of motion of both sexes (*n* = 20) in the four helmet conditions when pointing towards the orthogonal targets.

	No NVG						NVG					
	No HBS		HBS		Main effect – sex	Main effect – HBS	No HBS		HBS		Main effect – sex	Main effect – HBS
	M	F	M	F			M	F	M	F		
Up (extension)	40.8 (8.4)	35.8 (9.3)	34.2 (8.1)	27.6 (7.9)	0.015 (0.903)	0.479 (0.494)	39.8 (5.6)	36.5 (8.8)	38.6 (9.7)	35.5 (8.6)	1.287 (0.265)	0.156 (0.695)
Right (rotation)	**73.1 (8.2)**	**65.4 (9.4)**	**68.4 (10.5)**	**60.0 (11.8)**	**6.378 (0.016)**	2.522 (0.121)	**76.2 (11.2)**	**65.0 (15.6)**	**72.0 (12.1)**	**59.3 (9.6)**	**8.689 (0.006)**	1.536 (0.224)
Down (flexion)	**44.0 (10.4)**	**36.7 (10.8)**	**29.7 (12.9)**	**18.4 (9.0)**	**7.266 (0.011)**	**22.332 (<0.001)**	**41.8 (4.4)**	**34.0 (13.2)**	**35.1 (15.9)**	**23.8 (10.6)**	**5.947 (0.020)**	**4.581 (0.040)**
Left (rotation)	54.1 (7.3)	50.9 (9.4)	53.0 (11.5)	46.3 (7.7)	2.942 (0.095)	0.973 (0.331)	61.6 (9.0)	54.9 (13.4)	60.8 (10.3)	53.1 (11.5)	3.813 (0.059)	0.133 (0.717)

*Note*: Data are shown as means (SD) and statistical results are shown as *F*‐value (*P*‐value).

The bold data just highlight where there was a main effect and the F and P value for the main effect is given in the table already. There are no other p‐values that need ot be added to the table.

### 3.2 Phase A sex differences

There was no significant difference in muscle activation between sexes during the no helmet condition in all directions (Supplementary Table S2). For the four helmet‐wearing conditions (scalene muscles presented in Tables 2 and 3, with the remaining muscle data in Supplementary Tables S3–S8), females had higher muscle activity than males when looking Right [right Sca: 1.27 (0.47) and 1.07 (0.37) vs. 0.86 (0.38) and 0.88 (0.36), *P* = 0.027, Table 2; left SpC: 1.60 (0.69) and 1.16 (0.55) vs. 0.97 (0.33) and 1.05 (0.41), *P* = 0.030, Supplementary Table S8)] Left and up [left Sca: 1.07 (0.17) and 1.20 (0.26) vs. 0.87 (0.14) and 0.98 (0.23), *P* = 0.004, Table 3], at Centre [right SCM: 1.05 (0.40) and 1.55 (0.78) vs. 0.88 (0.26) and 1.02 (0.39), *P* = 0.041, Supplementary Table S3; right SpC: 1.60 (0.52) and 1.34 (0.65) vs. 1.09 (0.48) and 1.01 (0.31), *P* = 0.035, Supplementary Table S7], and Down [left SpC: 2.03 (0.41) and 1.50 (0.95) vs. 1.25 (0.46) and 1.09 (0.41), *P* = 0.008, Supplementary Table S8].

**TABLE 2:** Normalised (to head only) right scalene muscle activation levels of both sexes (*n* = 20) in the four helmet conditions when pointing towards the nine environmental targets.

Right scalene	Helmet						Helmet plus NVG					
	No HBS		HBS		Main effect – sex	Main effect – HBS	No HBS		HBS		Main effect – sex	Main effect – HBS
	M	F	M	F			M	F	M	F		
Centre	0.87 (0.24)	1.22 (0.43)	1.04 (0.46)	1.16 (0.39)	3.550 (0.068)	0.195 (0.661)	0.83 (0.26)	1.35 (0.52)	1.02 (0.39)	0.99 (0.22)	2.667 (0.154)	0.256 (0.631)
Up	1.24 (0.41)	1.09 (0.42)	1.07 (0.24)	1.29 (0.96)	0.032 (0.860)	0.007 (0.933)	**1.48 (0.56)**	**1.67 (0.89)**	**1.20 (0.45)**	**1.03 (0.53)**	0.004 (0.948)	**4.635 (0.039)**
Right and up	0.93 (0.22)	1.15 (0.32)	0.99 (0.20)	1.33 (0.82)	3.545 (0.068)	0.654 (0.424)	1.01 (0.27)	1.24 (0.27)	1.02 (0.16)	1.04 (0.25)	2.520 (0.122)	1.527 (0.225)
Right	**0.86 (0.38)**	**1.27 (0.47)**	**0.88 (0.36)**	**1.07 (0.37)**	**5.295 (0.027)**	0.499 (0.485)	1.06 (0.18)	0.96 (0.34)	1.32 (0.90)	1.07 (0.13)	1.032 (0.317)	1.182 (0.285)
Right and down	1.03 (0.19)	1.03 (0.31)	0.86 (0.30)	1.22 (0.57)	2.319 (0.137)	0.008 (0.929)	0.84 (0.26)	1.23 (0.52)	1.09 (0.46)	0.90 (0.33)	0.553 (0.462)	0.098 (0.757)
Down	1.12 (0.39)	1.15 (0.48)	1.11 (0.34)	1.27 (0.49)	0.504 (0.482)	0.155 (0.696)	1.09 (0.21)	1.18 (0.46)	1.12 (0.36)	0.94 (0.39)	0.153 (0.698)	0.703 (0.408)
Left and down	1.23 (0.88)	1.17 (0.73)	0.98 (0.46)	0.98 (0.48)	0.022 (0.884)	1.071 (0.308)	**1.75 (1.02)**	**1.72 (0.69)**	**0.93 (0.47)**	**1.00 (0.39)**	0.010 (0.920)	**11.884 (0.002)**
Left	1.00 (0.36)	1.04 (0.38)	1.03 (0.46)	1.22 (0.77)	0.476 (0.495)	0.388 (0.537)	1.83 (1.09)	1.56 (0.74)	1.27 (0.65)	1.28 (0.72)	0.233 (0.633)	2.484 (0.125)
Left and up	1.29 (0.98)	0.91 (0.31)	1.12 (0.72)	0.85 (0.45)	2.444 (0.127)	0.298 (0.588)	**1.95 (0.65)**	**1.18 (0.51)**	**1.20 (0.41)**	**1.03 (0.60)**	**6.157 (0.019)**	**5.901 (0.021)**

*Note*: Data are shown as means (SD) and statistical results are shown as *F*‐value (*P*‐value).

**TABLE 3:** Normalised (to head only) left scalene muscle activation levels of both sexes (*n* = 20) in the four helmet conditions when pointing towards the nine environmental targets.

Left scalene	Helmet						Helmet plus NVG					
	No HBS		HBS		Main effect – Sex	Main effect – HBS	No HBS		HBS		Main effect – sex	Main effect – HBS
	M	F	M	F			M	F	M	F		
Centre	0.95 (0.29)	0.91 (0.24)	1.09 (0.48)	0.96 (0.17)	0.668 (0.419)	0.853 (0.362)	0.88 (0.28)	1.02 (0.22)	0.97 (0.36)	0.86 (0.22)	0.001 (0.971)	0.941 (0.370)
Up	**1.36 (0.57)**	**0.89 (0.15)**	**1.25 (0.53)**	**1.02 (0.52)**	**5.340 (0.027)**	0.002 (0.968)	**1.63 (0.59)**	**1.57 (0.53)**	**1.20 (0.48)**	**1.06 (0.50)**	0.353 (0.556)	**7.238 (0.011)**
Right and up	0.77 (0.38)	0.63 (0.26)	0.83 (0.59)	0.64 (0.29)	1.693 (0.201)	0.085 (0.772)	1.69 (0.98)	1.21 (0.71)	1.15 (0.44)	0.87 (0.35)	3.070 (0.089)	4.098 (0.051)
Right	0.72 (0.32)	0.90 (0.45)	0.95 (0.53)	0.93 (0.41)	0.325 (0.572)	0.730 (0.399)	1.62 (0.73)	1.23 (0.53)	1.09 (0.51)	1.10 (0.51)	0.987 (0.328)	2.910 (0.098)
Right and down	0.96 (0.28)	0.93 (0.52)	0.99 (0.50)	0.74 (0.56)	0.878 (0.355)	0.284 (0.597)	**1.61 (0.77)**	**1.57 (0.88)**	**1.31 (0.56)**	**0.76 (0.51)**	1.655 (0.207)	**5.870 (0.021)**
Down	1.16 (0.40)	1.18 (0.49)	1.08 (0.34)	1.11 (0.23)	0.037 (0.849)	0.382 (0.540)	1.27 (0.36)	1.71 (0.96)	1.35 (0.76)	1.04 (0.13)	0.115 (0.737)	1.954 (0.171)
Left and down	1.18 (0.61)	1.09 (0.49)	0.90 (0.39)	0.99 (0.35)	0.000 (0.989)	1.676 (0.204)	0.78 (0.23)	1.26 (0.57)	0.96 (0.35)	0.88 (0.25)	2.590 (0.117)	0.580 (0.452)
Left	1.25 (0.85)	1.01 (0.17)	0.95 (0.23)	1.36 (0.74)	0.180 (0.674)	0.025 (0.874)	1.24 (0.32)	1.28 (0.43)	1.18 (0.46)	1.06 (0.39)	0.072 (0.790)	1.122 (0.297)
Left and up	**0.87 (0.14)**	**1.07 (0.17)**	**0.98 (0.23)**	**1.20 (0.26)**	**9.565 (0.004)**	3.329 (0.077)	0.91 (0.24)	1.40 (0.41)	1.10 (0.56)	1.01 (0.18)	2.399 (0.131)	0.624 (0.435)

*Note*: Data are shown as means (SD) and statistical results are shown as *F*‐value (*P*‐value).

Males had higher muscle activity than females when looking Left and up [right Sca: 1.95 (0.65) and 1.20 (0.41) vs. 1.18 (0.51) and 1.03 (0.60), *P* = 0.019, Table 2], Up [left Sca: 1.36 (0.57) and 1.25 (0.53) vs. 0.89 (0.15) and 1.02 (0.52), *P* = 0.027, Table 3; left SCM: 0.76 (0.38) and 0.94 (0.54) vs. 0.51 (0.21) and 0.64 (0.28), *P* < 0.001, Supplementary Table S4], Left [left UT: 1.23 (0.58) and 1.32 (0.41) vs. 1.06 (0.24) and 0.95 (0.20), *P* = 0.044, Supplementary Table S6] and Right and up [left SCM: 0.76 (0.38) and 0.94 (0.54) vs. 0.51 (0.21) and 0.64 (0.28), *P* = 0.032, Supplementary Table S4].

The sex differences were more prominent in the helmet without NVG condition (lighter) than the NVG condition; and were more prominent when the targets were above horizontal (Up, Right and up, Left and up) than those below (Down, Right and down, Left and down).

### 3.3 Phase A HBS effects

Applying the HBS showed an overall trend of reducing muscle activities, with the reduction more likely to happen when using NVG (heavier). The only two significant differences found in the without‐NVG conditions were the SpC muscles when looking towards Left and up [right SpC: 0.94 (0.16) and 0.80 (0.31) vs. 1.09 (0.30) and 1.13 (0.58), *P* = 0.046, Supplementary Table S7] and Left and down [left SpC: 0.75 (0.29) and 0.75 (0.30) vs. 1.23 (0.61) and 1.30 (0.79), *P* = 0.005, Supplementary Table S8]. Except for the right UT muscle when looking towards Right [1.14 (0.55) and 1.48 (0.80) vs. 0.96 (0.15) and 0.99 (0.15), *P* = 0.050, Supplementary Table S5] and Right and down [1.11 (0.22) and 1.26 (0.66) vs. 0.95 (0.88) and 0.91 (0.07), *P* = 0.032, Supplementary Table S5], all the other significances found demonstrated a reduction in muscle activities with the application of the HBS.

### 3.4 Phase B sex differences

Compared to the baseline, both females and males showed a decrease in RMS EMG during post‐test MVICs after wearing the helmet for 45 min (Table 4). There was a significant main effect of sex in RMS EMG during post‐test MVICs with females exhibiting a greater reduction than males in the right SCM [0.82 (0.09) vs. 0.93 (0.06) and 0.84 (0.11) vs. 0.85 (0.11), *P* = 0.037; no HBS and HBS conditions, respectively] and the right SpC [0.68 (0.20) vs. 0.85 (0.19) and 0.84 (0.21) vs. 0.93 (0.14), *P* = 0.032]. For the median frequency (Table 4), there was a significant main effect of sex on the left SpC muscle but this was not significant on *post hoc* analysis [no HBS, female vs. male: 0.88 (0.16) vs. 0.96 (0.07), *P* = 0.127; HBS, female vs. male: 1.07 (0.13) vs. 0.94 (0.14), *P* = 0.156].

**TABLE 4:** Normalised (to pre‐test MVIC baseline) RMS EMG and median frequency of all the tested muscles of both sexes (*n* = 20) in the four helmet conditions.

	Root‐mean‐square EMG						Median frequency					
	No HBS		HBS		Main effect – sex	Main effect – HBS	No HBS		HBS		Main effect – sex	Main effect – HBS
	M	F	M	F			M	F	M	F		
Right SCM	**0.93 (0.06)**	**0.82 (0.09)**	**0.85 (0.11)**	**0.84 (0.11)**	**4.690 (0.037)**	0.792 (0.380)	1.04 (0.06)	1.06 (0.05)	1.01 (0.04)	1.03 (0.04)	2.057 (0.160)	2.854 (0.100)
Right Sca	0.81 (0.12)	0.82 (0.15)	0.94 (0.19)	0.83 (0.17)	0.944 (0.338)	1.914 (0.175)	1.01 (0.07)	1.01 (0.06)	1.06 (0.12)	1.02 (0.06)	0.423 (0.519)	1.595 (0.215)
Right UT	0.85 (0.14)	0.69 (0.12)	0.84 (0.21)	0.81 (0.20)	3.290 (0.078)	0.926 (0.342)	1.03 (0.09)	1.00 (0.12)	0.97 (0.14)	1.03 (0.07)	0.155 (0.696)	0.112 (0.739)
Right SpC	**0.85 (0.19)**	**0.68 (0.20)**	**0.93 (0.14)**	**0.84 (0.21)**	**4.994 (0.032)**	**4.114 (0.050)**	1.01 (0.05)	1.00 (0.13)	0.99 (0.04)	1.01 (0.08)	0.069 (0.794)	0.006 (0.940)
Left SCM	0.87 (0.13)	0.84 (0.16)	0.86 (0.12)	0.84 (0.14)	0.203 (0.655)	0.003 (0.954)	1.02 (0.07)	1.02 (0.04)	0.96 (0.10)	1.02 (0.09)	1.331 (0.256)	1.524 (0.225)
Left Sca	0.88 (0.13)	0.85 (0.17)	0.87 (0.11)	0.72 (0.19)	3.577 (0.067)	1.954 (0.171)	**0.98 (0.12)**	**0.99 (0.03)**	**1.01 (0.05)**	**1.06 (0.05)**	1.603 (0.214)	**4.087 (0.050)**
Left UT	0.79 (0.20)	0.80 (0.14)	0.85 (0.20)	0.86 (0.23)	0.021 (0.887)	1.142 (0.292)	**0.98 (0.09)**	**0.92 (0.12)**	**1.01 (0.07)**	**1.04 (0.06)**	0.245 (0.623)	**8.723 (0.006)**
Left SpC	0.82 (0.13)	0.81 (0.19)	0.89 (0.18)	0.79 (0.18)	1.295 (0.263)	0.195 (0.661)	**0.96 (0.07)**	**0.88 (0.16)**	**0.94 (0.14)**	**1.07 (0.13)**	**4.294 (0.048)**	**9.418 (0.005)**

*Note*: Data are shown as means (SD) and statistical results are shown as *F*‐value (*P*‐value).

**FIGURE 2:**
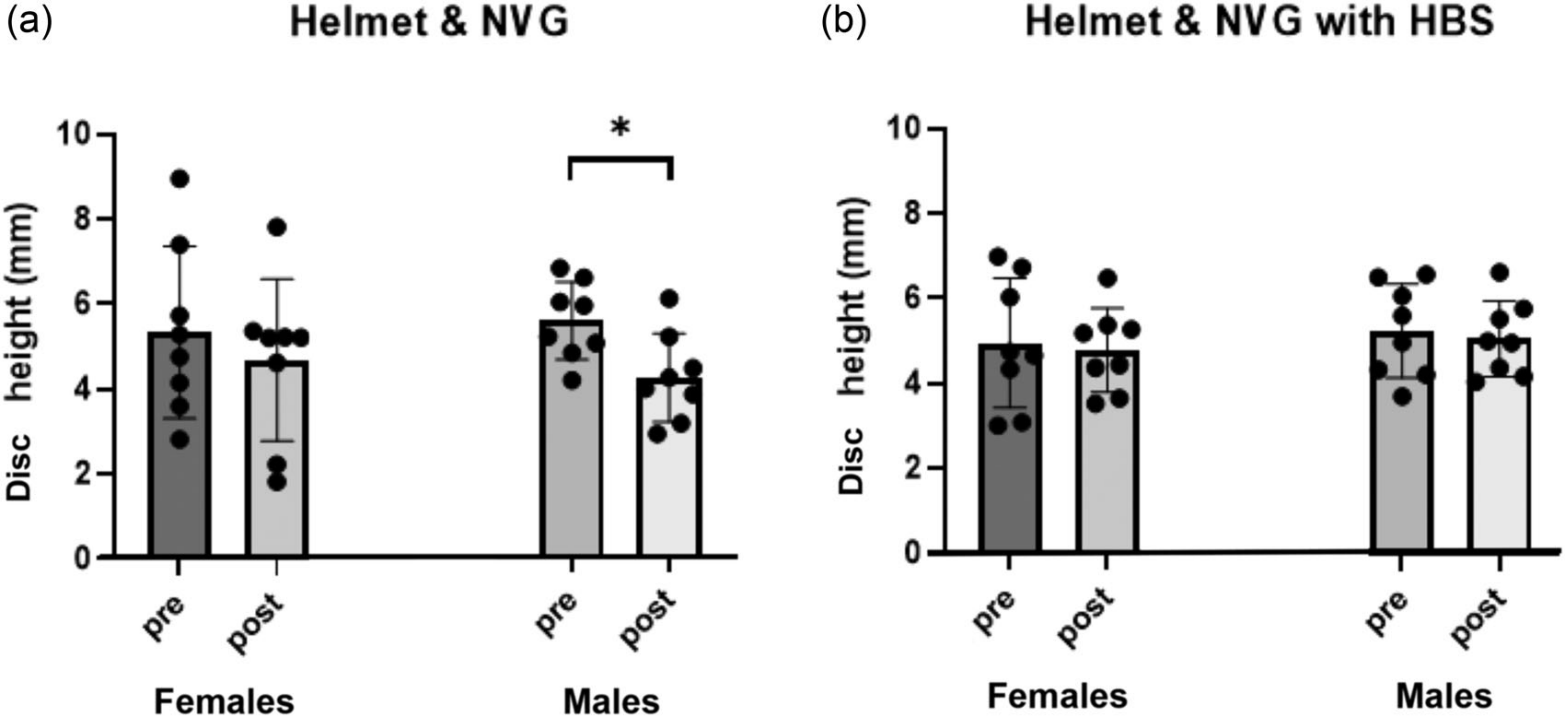
Ultrasound‐measured anterior intervertebral disc height (*n* = 8 for each group) measured without (a) and with HBS (b). *Significant difference between pre‐ and post‐45 min of continuous helmet wearing, *P* < 0.001.

Comparing to the baseline, cervical disc height was significantly reduced after 45 min in the NVG condition in males [*n* = 8, 5.7 mm (1.4) vs. 4.4 mm (1.5), *P* < 0.001], but was unchanged in females [*n* = 8, 5.0 mm (2.0) vs. 4.7 mm (2.2), *P* = 0.605, Figure 2a].

### 3.5 Phase B HBS effects

With the HBS activated, the post‐test RMS EMG of the right SpC muscle [0.93 (0.14) and 0.84 (0.21) vs. 0.85 (0.19) and 0.68 (0.20), *P* = 0.050] was significantly higher than those without HBS condition (Table 4). The median frequency of the left scalene [1.01 (0.05) and 1.06 (0.05) vs. 0.98 (0.12) and 0.99 (0.03), *P* = 0.050), left UT (1.01 (0.07) and 1.04 (0.06) vs. 0.98 (0.09) and 0.92 (0.12), *P* = 0.006] and left SpC [0.94 (0.14) and 1.07 (0.13) vs. 0.96 (0.07) and 0.88 (0.16), *P* = 0.005] muscles was significantly higher in the HBS condition than the without HBS condition (Table 4).

There were no changes in disc height for either sex [*n* = 8, male: 5.0 (1.3) vs. 5.2 (1.4) mm, *P* = 0.604; *n* = 8, female: 4.8 (1.1) vs. 4.9 (1.7) mm, *P* = 0.947] after 45 min of wearing a helmet with the HBS activated (Figure 2b).

### 3.6 Correlation with body height and weight

No correlations were found between body height or weight and muscle activity for non‐HBS conditions (helmet‐only and helmet plus NVG) in Phase A. Similarly, no correlations were observed between body dimensions and the pre–post ratio of RMS EMG, median frequency and cervical disc height in Phase B. The coefficient of determination was low for both EMG‐related measurements (*R* ^2^ <0.01 to 0.22) and ultrasound‐measured disc height (*R* ^2^ 0.07 to 0.23).

## 4 DISCUSSION

The results of the current study confirmed our hypothesis that females and males demonstrate different neuromuscular responses to wearing a helmet. While for many of the muscle activities recorded, there were no differences between sexes, when differences were apparent, females typically exhibited higher muscular activities to support the weight of the helmet compared to males. This was evident during the acute dynamic movement tasks in Phase A and further supported by findings from Phase B, indicating more fatigue‐related changes in females compared to males after prolonged wear. In contrast, males may rely more on passive spinal structures to support the additional weight of a helmet compared to females, as indicated by the decrease in cervical disc height during prolonged helmet wear.

Although we standardised the seating height for each participant, the range of motions for participants when looking towards the environmental targets was different, with females having a more limited range of motion than males. While this could be attributed to helmet wearing, the females already had lower ranges than males when not wearing helmets. This may result from the physiological or anatomical differences in the head or neck affecting an individual's eye–head coordination (for review see Proudlock & Gottlob, 2007) that we were unable to quantify in this study. Interestingly, it is reported that while the neck ranges of motion are similar between sexes in the 20s age group, females in their 30s and 40s showed small (5–10 deg), but significantly higher mobility than males in the sagittal and horizontal plane movements (meta‐analysis by Pan et al., 2018). Despite exhibiting smaller ranges of motion in females, the differences were often small and the movement degrees were within the expected range and representative of the actual flying scenario. Given the lower range of motion, the greater muscle activities (Phase A) and fatigue (Phase B) found in females are unlikely to be the direct result of females simply having greater head/neck movement than their male counterparts.

Our EMG results are similar to the findings reported by Harrison et al. (2016) where 16 male pilots performed movements as they normally would while flying with either NVGs or NVGs with counterweight attached. Compared to the head‐only condition, both NVG conditions increased neck muscle EMG intensity from 150 to 350% in most of the movement directions. While previous research (Harrison et al., 2016) suggested a significant difference between the right and left sides, the current study did not show a clear trend in this regard across conditions despite using similar environmental settings and movement conditions. It has been suggested that the right trapezius muscle showed elevated blood oxygenation and volume, measured by near‐infrared spectroscopy, among male pilots in a simulated flight with NVG attached (Harrison et al., 2007). It may be possible that these lateral differences are more prominent in pilots than in our study population since during flight at night a large proportion of the time is spent with mild neck flexion (74.5%) and mild axial twisted posture (63.1%) (Forde et al., 2011) that could potentially develop habitual side‐differences in muscle activation.

Our findings indicate that the most prominent sex difference in Phase A was observed in the scalene muscles. The scalene muscles play a crucial role in both breathing and neck stability as they connect the cervical spine to the upper ribs. Previous studies have suggested that their activity is correlated with head posture and chronic neck pain (Kang et al., 2018). Falla et al. (2004, 2003) investigated the fatigue pattern of the Sca muscle in individuals with neck pain compared to asymptomatic individuals. Their results indicated that individuals with neck pain exhibited increased fatiguability, particularly at lower force (<50% MVIC) conditions, compared to asymptomatic individuals, with it suggested that scalene muscle activity could serve as an objective measurement of muscular dysfunction in individuals with neck pain, and interventions targeting this muscle may be beneficial in reducing pain. To the best of our knowledge, there are no reports analysing the biomechanical differences between sexes in the scalene muscles. Based on our results, we suggest that this could be an area of focus for future measurements in neck biomechanics between sexes.

There was no significant difference in the baseline anterior cervical disc height between sexes, and our reported values were similar to those reported by Zheng et al. (2016, 2014). It is intriguing to consider that muscle fatigue may reduce the capacity to withstand a load, leading to more force being transmitted to passive structures (Kazemi et al., 2023; Raabe & Chaudhari, 2018; Rehan Youssef et al., 2009). However, we did not observe a reduced disc height in females despite greater levels of muscle activation and fatigue compared to males. The present study used a 45 min wear period; however, it is not uncommon for some military pilots to wear a helmet with NVG attached for more than an hour during sorties. With increased duration, it is likely that more prominent muscular fatigue along with further compression of the cervical intervertebral discs can occur.

We found no correlation between body height or weight with our biomechanical outcomes although this does not exclude other measures such as mass of the head or the strength of individual neck muscles having an impact. It is possible that given the difference in the head and neck morphology, the moment of inertia after helmet donning may differ between males and females. Although not designed to test this, the higher muscle activities observed in the current study could potentially result from a more mechanically disadvantaged position among female participants after donning a helmet, such as the centre of gravity of the helmet deviating from the centre of gravity of the head (Phillips & Petrofsky, 1986). Due to the relatively low number of females in the military, there is a lack of epidemiological studies regarding chronic neck problems in female pilots (Harrison et al., 2011). At present, no specific conditioning programs focus on neck muscles tailored for female pilots (Farrell et al., 2020; James et al., 2021). It is known that under a similar training load, females are more likely to suffer from musculoskeletal injuries (Nindl et al., 2016; Thomas et al., 2023). With an increasing number of female personnel in the military, consideration should be given to the need for tailored interventions that account for any potential sex‐related differences in aircrew. This current study warrants the need to emphasise and further investigate potential sex differences, either in flight or after conditioning programmes, in neck biomechanical responses among pilots in the future.

The findings from this study suggest that the use of HBS may be beneficial in reducing helmet‐related neck muscle loads, especially among females. These results imply that implementing external support, such as HBS or a similar spring‐based system (Dibblee et al., 2015), could be a proactive measure in preventing neck problems among pilots. It is reasonable to hypothesise that with longer flight duration, the beneficial effects (such as reducing muscular fatigue and preventing intervertebral disc compression) of HBS will be more prominent for both sexes than our current 45 min settings and could potentially reduce chronic neck pain prevalence among pilots after long‐term use. However, our healthy non‐pilot participants may not be representative of military pilots and do not have experience of chronic helmet‐wear and any potential effects it may have. Future study of pilots is warranted to fully understand the effectiveness of HBS in reducing helmet‐associated muscular fatigue or spinal changes.

There are limitations of our study that prevent further interpretation of the results. The sample size of the study was small, and care should be taken when extrapolating the result to the general population or pilots regarding neck pain. We were unable to record force during the pre‐ and post‐test MVICs and, therefore, were unable to indicate whether muscle strength decreases after prolonged helmet wear, although many of the participants reported subjective weakness during the post‐test MVIC. Our ultrasonography methodology only allowed measurement of the anterior distance of the intervertebral disc in the sagittal plane and the measurement accuracy could be affected by the distortion of image resulted from the variation in the speed of sound throughout the tissues (Bland et al., 2017). Due to the limited space available after putting on a helmet and attaching EMG sensors bilaterally, we could not measure the exact cervical position during the ultrasound scan. It is possible that a shift in head and neck angle can influence our measurement of the disc height, although to minimise this we ensured a standardised position for the head during the measurement. We did not control the time of the day when conducting the experiment due to logistical reasons and the availability of participants. The diurnal change of the cervical disc height may affect the pre‐test baseline, but we would expect that the observed difference after 45 min predominantly resulted from helmet‐wearing. Our application of surface EMG could only target superficial muscles. There could be changes to the deep muscles (e.g., multifidus) that cannot be detected using this approach (Choi et al., 2021). Lastly, it is not known whether the menstrual cycle influences the reported outcome of neck neuromuscular activities and spinal disc height or affects the biomechanical responses to changing the helmet loading through HBS.

### 4.1 Conclusion

In summary, our study suggests that females and males demonstrate different muscle activity patterns and changes in spinal structures when manipulating the neck external load applied through a helmet. These results indicate the potential usefulness for preventing musculoskeletal injuries through mechanically unloading the weight of helmets and imply that different physical training or preventative regimes targeting distinct structures could be considered for pilots of different sexes.

## AUTHOR CONTRIBUTIONS

All the experiments were conducted in the laboratory at the Centre for Human & Applied Physiological Sciences, King's College London. Conception or design of the work: Nai‐Hao Yin, Irene Di Giulio, Peter D. Hodkinson, Federico Formenti, and Ross D. Pollock. Acquisition, analysis, or interpretation of data for the work: Nai‐Hao Yin, Irene Di Giulio, Peter D. Hodkinson, Federico Formenti, and Ross D. Pollock. Drafting of the work or revising it critically for important intellectual content: Nai‐Hao Yin, Irene Di Giulio, Peter D. Hodkinson, Federico Formenti, and Ross D. Pollock. All authors have approved the final version of the manuscript and agree to be accountable for all aspects of the work in ensuring that questions related to the accuracy or integrity of any part of the work are appropriately investigated and resolved. All persons designated as authors qualify for authorship, and all those who qualify for authorship are listed.

## CONFLICT OF INTEREST

The authors declare no conflicts of interest.

## Supporting information

Tables S1–S8.

## Data Availability

The data that support the findings of this study are available in the supplementary material of this article.
