# Supplementary material for: Sex differences in cervical disc height and neck muscle activation during manipulation of external load from helmets
Source: Exp Physiol. 2024 Aug 9;109(10):1728–38. doi: 10.1113/EP091996 (PMC11442774; doi:10.1113/EP091996)
Supplement: Supplementary file 1 — Tables S1–S8. [file EPH-109-1728-s001.docx]

**Supporting Information**

**Table S1.** Range of motion of both sexes in the four helmet conditions when pointing towards the diagonal targets. Data are shown as mean (standard deviation) and statistical results are shown as F-value (p-value).

|  | No NVG | | | | | | NVG | | | | | |
| --- | --- | --- | --- | --- | --- | --- | --- | --- | --- | --- | --- | --- |
|  | No HBS | | HBS | | Main effect - Sex | Main effect - HBS | No HBS | | HBS | | Main effect - Sex | Main effect - HBS |
|  | M | F | M | F |  |  | M | F | M | F |  |  |
| **Right & Up** (rotation) | **53.1 (10.2)** | **46.2 (10.5)** | **47.7 (6.6)** | **40.5 (10.9)** | **5.351 (.027)** | 3.259 (.079) | **54.0 (8.1)** | **45.8 (11.6)** | **54.4 (7.3)** | **44.4 (11.3)** | **8.242 (.007)** | 0.022 (.882) |
| (extension) | **24.1 (5.9)** | **23.6 (7.0)** | **19.9 (6.7)** | **16.5 (8.5)** | 0.731 (.398) | **6.423 (.016)** | **28.2 (3.9)** | **20.8 (7.6)** | **26.3 (9.3)** | **23.4 (8.1)** | **4.175 (.049)** | 0.021 (.887) |
| **Right & Down** (rotation) | **75.7 (8.8)** | **66.0 (10.3)** | **59.9 (18.8)** | **52.7 (9.1)** | **4.590 (.039)** | **13.456 (<.001)** | **75.3 (8.6)** | **68.8 (12.3)** | **63.0 (14.1)** | **54.6 (9.2)** | 4.003 (.054) | **12.472 (.001)** |
| (flexion) | **36.7 (9.0)** | **27.8 (7.6)** | **22.0 (12.6)** | **15.0 (8.1)** | **6.758 (.014)** | **20.173 (<.001)** | **38.7 (8.9)** | **26.6 (8.5)** | **25.5 (12.1)** | **18.1 (11.7)** | **7.815 (.009)** | **9.704 (.004)** |
| **Left & Down** (rotation) | **75.0 (13.0)** | **67.5 (14.5)** | **63.8 (12.7)** | **53.8 (14.8)** | 4.016 (.053) | **8.157 (.007)** | **80.8 (12.9)** | **69.6 (13.6)** | **66.3 (15.1)** | **58.2 (14.2)** | **4.374 (.044)** | **7.988 (.008)** |
| (flexion) | **37.1 (7.7)** | **31.0 (5.4)** | **22.3 (10.6)** | **17.5 (10.9)** | 3.664 (.064) | **24.865 (<.001)** | **43.3 (14.7)** | **32.2 (8.6)** | **28.2 (13.7)** | **16.9 (10.1)** | **7.932 (.008)** | **14.587 (<.001)** |
| **Left & Up** (rotation) | **44.3 (6.3)** | **42.3 (6.5)** | **42.4 (5.0)** | **36.3 (4.2)** | **5.340 (.027)** | **4.749 (.036)** | **49.8 (10.3)** | **41.0 (10.1)** | **46.4 (6.0)** | **42.7 (9.9)** | **4.137 (.050)** | 0.077 (.783) |
| (extension) | 21.7 (8.0) | 25.0 (6.4) | 22.7 (5.1) | 19.9 (7.4) | 0.011 (.918) | 0.873 (.165) | 25.2 (4.4) | 21.6 (5.1) | 21.8 (6.4) | 23.1 (8.4) | 0.313 (.580) | 0.190 (.666) |

**Table S2**. Normalised (to pre-test MVIC) muscle activation levels of both sexes in the no helmet condition when pointing towards the nine environmental targets. Data are shown as mean (standard deviation).

| **Right** | **Sternocleidomastoid** | | | **Scalenes** | | | **Upper Trapezius** | | | **Splenius Capitis** | | |
| --- | --- | --- | --- | --- | --- | --- | --- | --- | --- | --- | --- | --- |
|  | Male | Female | p-value | Male | Female | p-value | Male | Female | p-value | Male | Female | p-value |
| Centre | 0.01 (0.01) | 0.01 (0.01) | 0.370 | 0.01 (0.01) | 0.02 (0.02) | 0.609 | 0.02 (0.02) | 0.01 (0.01) | 0.609 | 0.04 (0.04) | 0.03 (0.03) | 0.312 |
| Up | 0.01 (0.01) | 0.03 (0.02) | 0.067 | 0.02 (0.01) | 0.04 (0.04) | 0.185 | 0.01 (0.01) | 0.01 (0.01) | 0.313 | 0.06 (0.06) | 0.06 (0.08) | 0.992 |
| Right & Up | 0.01 (0.01) | 0.02 (0.01) | 0.694 | 0.02 (0.02) | 0.02 (0.01) | 0.887 | 0.01 (0.01) | 0.01 (0.01) | 0.165 | 0.13 (0.09) | 0.10 (0.11) | 0.537 |
| Right | 0.02 (0.03) | 0.03 (0.04) | 0.518 | 0.08 (0.20) | 0.03 (0.03) | 0.462 | 0.01 (0.01) | 0.01 (0.01) | 0.232 | 0.21 (0.20) | 0.20 (0.21) | 0.928 |
| Right & Down | 0.03 (0.03) | 0.02 (0.01) | 0.742 | 0.03 (0.02) | 0.03 (0.02) | 0.541 | 0.01 (0.01) | 0.01 (0.01) | 0.129 | 0.11 (0.10) | 0.11 (0.11) | 0.931 |
| Down | 0.01 (0.02) | 0.02 (0.01) | 0.545 | 0.02 (0.01) | 0.03 (0.03) | 0.243 | 0.01 (0.01) | 0.01 (0.01) | 0.170 | 0.04 (0.03) | 0.04 (0.03) | 0.915 |
| Left & Down | 0.13 (0.14) | 0.11 (0.09) | 0.751 | 0.06 (0.07) | 0.05 (0.04) | 0.949 | 0.01 (0.01) | 0.01 (0.01) | 0.869 | 0.07 (0.07) | 0.05 (0.05) | 0.781 |
| Left | 0.11 (0.08) | 0.08 (0.08) | 0.422 | 0.05 (0.06) | 0.04 (0.03) | 0.670 | 0.02 (0.02) | 0.01 (0.01) | 0.579 | 0.05 (0.04) | 0.05 (0.03) | 0.834 |
| Left & Up | 0.07 (0.06) | 0.08 (0.08) | 0.794 | 0.04 (0.04) | 0.06 (0.07) | 0.500 | 0.01 (0.01) | 0.01 (0.01) | 0.683 | 0.06 (0.04) | 0.05 (0.03) | 0.615 |
|  |  |  |  |  |  |  |  |  |  |  |  |  |
| **Left** | **Sternocleidomastoid** | | | **Scalenes** | | | **Upper Trapezius** | | | **Splenius Capitis** | | |
|  | Male | Female | p-value | Male | Female | p-value | Male | Female | p-value | Male | Female | p-value |
| Centre | 0.01 (0.01) | 0.01 (0.01) | 0.349 | 0.01 (0.01) | 0.01 (0.01) | 0.858 | 0.02 (0.02) | 0.01 (0.01) | 0.789 | 0.03 (0.02) | 0.03 (0.03) | 0.896 |
| Up | 0.01 (0.01) | 0.04 (0.03) | 0.069 | 0.02 (0.01) | 0.02 (0.01) | 0.475 | 0.01 (0.02) | 0.01 (0.01) | 0.711 | 0.05 (0.04) | 0.04 (0.04) | 0.897 |
| Right & Up | 0.09 (0.07) | 0.12 (0.10) | 0.484 | 0.08 (0.09) | 0.06 (0.04) | 0.427 | 0.02 (0.02) | 0.02 (0.02) | 0.598 | 0.08 (0.09) | 0.06 (0.05) | 0.483 |
| Right | 0.14 (0.13) | 0.14 (0.09) | 0.973 | 0.13 (0.23) | 0.07 (0.05) | 0.375 | 0.03 (0.04) | 0.03 (0.02) | 0.755 | 0.05 (0.04) | 0.07 (0.06) | 0.410 |
| Right & Down | 0.10 (0.13) | 0.12 (0.09) | 0.721 | 0.05 (0.08) | 0.05 (0.04) | 0.912 | 0.02 (0.02) | 0.02 (0.01) | 0.766 | 0.05 (0.03) | 0.09 (0.11) | 0.270 |
| Down | 0.01 (0.01) | 0.01 (0.00) | 0.827 | 0.01 (0.01) | 0.01 (0.01) | 0.714 | 0.01 (0.02) | 0.01 (0.01) | 0.829 | 0.05 (0.04) | 0.04 (0.04) | 0.609 |
| Left & Down | 0.03 (0.04) | 0.02 (0.01) | 0.341 | 0.04 (0.04) | 0.02 (0.01) | 0.332 | 0.02 (0.02) | 0.02 (0.01) | 0.803 | 0.14 (0.13) | 0.12 (0.08) | 0.686 |
| Left | 0.01 (0.02) | 0.01 (0.01) | 0.744 | 0.03 (0.04) | 0.02 (0.01) | 0.474 | 0.01 (0.02) | 0.02 (0.01) | 0.737 | 0.12 (0.12) | 0.08 (0.06) | 0.328 |
| Left & Up | 0.01 (0.01) | 0.01 (0.01) | 0.295 | 0.02 (0.01) | 0.01 (0.01) | 0.740 | 0.01 (0.02) | 0.01 (0.01) | 0.833 | 0.13 (0.12) | 0.08 (0.06) | 0.223 |

**Table S3**. Normalised (to head only) right sternocleidomastoid muscle activation levels of both sexes in the four helmet conditions when pointing towards the nine environmental targets. Data are shown as mean (standard deviation) and statistical results are shown as F-value (p-value).

| **Right**  **SCM** | Helmet | | | | | | Helmet plus NVG | | | | | |
| --- | --- | --- | --- | --- | --- | --- | --- | --- | --- | --- | --- | --- |
|  | No HBS | | HBS | | Main effect - Sex | Main effect - HBS | No HBS | | HBS | | Main effect - Sex | Main effect - HBS |
|  | M | F | M | F |  |  | M | F | M | F |  |  |
| Centre | **0.88 (0.26)** | **1.05 (0.40)** | **1.02 (0.39)** | **1.55 (0.78)** | **4.510 (.041)** | **3.764 (.061)** | 0.89 (0.34) | 1.47 (0.72) | 1.00 (0.40) | 1.03 (0.30) | 3.142 (.127) | 0.748 (.420) |
| Up | 1.26 (0.45) | 1.04 (0.39) | 1.16 (0.51) | 1.34 (0.70) | 0.010 (.919) | 0.366 (.549) | 1.34 (0.41) | 1.61 (0.99) | 1.42 (0.92) | 1.35 (0.80) | 0.123 (.728) | 0.109 (.743) |
| Right & Up | 0.88 (0.11) | 1.09 (0.40) | 1.01 (0.32) | 1.30 (0.57) | 4.009 (.053) | 1.976 (.169) | 1.00 (0.14) | 1.26 (0.63) | 0.95 (0.12) | 0.94 (0.21) | 1.178 (.286) | 2.743 (.107) |
| Right | 0.98 (0.36) | 1.05 (0.32) | 1.03 (0.33) | 1.21 (0.42) | 1.246 (.272) | 0.939 (.339) | **1.15 (0.24)** | **1.12 (0.25)** | **0.88 (0.26)** | **0.93 (0.31)** | 0.028 (.868) | **6.537 (.016)** |
| Right & Down | 1.09 (0.56) | 1.03 (0.38) | 0.87 (0.39) | 1.14 (0.32) | 0.632 (.432) | 0.198 (.659) | 0.84 (0.31) | 1.16 (0.43) | 0.80 (0.39) | 0.90 (0.22) | 2.903 (.099) | 1.512 (.229) |
| Down | 1.37 (0.85) | 1.35 (0.72) | 1.26 (0.62) | 1.42 (0.56) | 0.094 (.760) | 0.008 (.930) | 1.26 (0.41) | 1.24 (0.75) | 1.17 (0.44) | 1.22 (0.79) | 0.008 (.930) | 0.075 (.786) |
| Left & Down | 1.10 (0.57) | 1.00 (0.39) | 0.95 (0.59) | 1.09 (0.61) | 0.016 (.900) | 0.028 (.869) | **1.37 (0.59)** | **1.29 (0.52)** | **0.87 (0.44)** | **0.97 (0.42)** | 0.005 (.946) | **6.374 (.017)** |
| Left | 0.95 (0.40) | 1.36 (0.73) | 1.01 (0.56) | 1.16 (0.71) | 1.984 (.168) | 0.107 (.745) | 1.91 (1.00) | 1.63 (0.97) | 1.25 (0.53) | 1.23 (0.84) | 0.274 (.604) | 3.320 (.078) |
| Left & Up | 1.30 (0.85) | 1.07 (0.44) | 1.07 (0.59) | 0.73 (0.29) | 2.257 (.142) | 2.252 (.142) | 1.57 (0.84) | 1.73 (1.12) | 1.10 (0.56) | 1.31 (0.91) | 0.387 (.539) | 2.190 (.149) |

**Table S4**. Normalised (to head only) left sternocleidomastoid muscle activation levels of both sexes in four helmet conditions when pointing towards the nine environmental targets. Data are shown as mean (standard deviation) and statistical results are shown as F-value (p-value).

| **Left**  **SCM** | Helmet | | | | | | Helmet plus NVG | | | | | |
| --- | --- | --- | --- | --- | --- | --- | --- | --- | --- | --- | --- | --- |
|  | No HBS | | HBS | | Main effect - Sex | Main effect - HBS | No HBS | | HBS | | Main effect - Sex | Main effect - HBS |
|  | M | F | M | F |  |  | M | F | M | F |  |  |
| Centre | 0.93 (0.24) | 0.92 (0.22) | 1.29 (0.64) | 1.07 (0.27) | 0.812 (.374) | 3.741 (.061) | 0.97 (0.25) | 1.06 (0.28) | 0.96 (0.24) | 0.84 (0.21) | 0.103 (.759) | 5.346 (.060) |
| Up | **1.32 (0.41)** | **0.74 (0.23)** | **1.16 (0.43)** | **0.76 (0.44)** | **15.067 (<.001)** | 0.297 (.590) | 1.64 (0.88) | 1.29 (0.57) | 1.21 (0.50) | 1.23 (0.86) | 0.686 (.414) | 0.870 (.358) |
| Right & Up | **0.76 (0.38)** | **0.51 (0.21)** | **0.94 (0.54)** | **0.64 (0.28)** | **4.991 (.032)** | 1.637 (.209) | 1.12 (0.61) | 1.05 (0.77) | 1.17 (0.69) | 0.87 (0.44) | 0.696 (.410) | 0.078 (.782) |
| Right | 0.73 (0.25) | 0.75 (0.21) | 0.93 (0.57) | 0.89 (0.34) | 0.002 (.967) | 1.964 (.170) | 1.18 (0.46) | 1.00 (0.32) | 1.05 (0.55) | 0.95 (0.52) | 0.695 (.411) | 0.338 (.565) |
| Right & Down | 0.85 (0.21) | 0.81 (0.48) | 0.75 (0.44) | 0.88 (0.87) | 0.070 (.793) | 0.008 (.927) | **1.12 (0.56)** | **1.23 (0.69)** | **0.83 (0.49)** | **0.55 (0.32)** | 0.248 (.622) | **7.441 (.010)** |
| Down | 0.93 (0.10) | 1.14 (0.29) | 1.26 (0.43) | 1.14 (0.30) | 0.228 (.636) | 2.788 (.104) | 1.30 (0.53) | 1.24 (0.38) | 1.08 (0.27) | 1.29 (0.74) | 0.218 (.644) | 0.250 (.621) |
| Left & Down | 1.19 (0.70) | 0.99 (0.36) | 0.77 (0.35) | 0.96 (0.40) | 0.001 (.982) | 2.053 (.161) | 0.95 (0.52) | 1.02 (0.37) | 0.95 (0.55) | 1.09 (0.79) | 2.590 (.117) | 0.580 (.452) |
| Left | 1.05 (0.45) | 1.00 (0.22) | 0.88 (0.21) | 1.43 (0.98) | 1.978 (.168) | 0.541 (.467) | 1.10 (0.25) | 1.05 (0.33) | 1.32 (0.75) | 1.05 (0.30) | 1.095 (.303) | 0.533 (.471) |
| Left & Up | 0.96 (0.11) | 1.04 (0.28) | 1.18 (0.62) | 0.97 (0.25) | 0.272 (.605) | 0.398 (.532) | 1.02 (0.16) | 1.37 (0.76) | 1.04 (0.20) | 1.04 (0.37) | 1.551 (.222) | 1.219 (.278) |

**Table S5**. Normalised (to head only) right upper trapezius muscle activation levels of both sexes in four helmet conditions when pointing towards the nine environmental targets. Data are shown as mean (standard deviation) and statistical results are shown as F-value (p-value).

| **Right**  **UT** | Helmet | | | | | | Helmet plus NVG | | | | | |
| --- | --- | --- | --- | --- | --- | --- | --- | --- | --- | --- | --- | --- |
|  | No HBS | | HBS | | Main effect - Sex | Main effect - HBS | No HBS | | HBS | | Main effect - Sex | Main effect - HBS |
|  | M | F | M | F |  |  | M | F | M | F |  |  |
| Centre | 0.79 (0.34) | 1.09 (0.39) | 0.90 (0.32) | 0.97 (0.28) | 2.799 (.103) | 0.000 (.982) | 1.00 (0.50) | 1.09 (0.36) | 0.84 (0.25) | 1.26 (0.67) | 2.591 (.168) | 0.055 (.824) |
| Up | 0.97 (0.28) | 1.08 (0.31) | 1.17 (0.65) | 1.22 (0.60) | 0.259 (.614) | 1.178 (.285) | 1.23 (0.46) | 1.07 (0.26) | 1.10 (0.41) | 1.29 (0.57) | 0.007 (.932) | 0.084 (.773) |
| Right & Up | 1.02 (0.22) | 1.07 (0.22) | 1.28 (0.58) | 1.20 (0.54) | 0.022 (.883) | 2.141 (.152) | 1.15 (0.32) | 0.99 (0.12) | 1.41 (0.82) | 1.11 (0.33) | 1.988 (.168) | 1.351 (.254) |
| Right | 0.91 (0.10) | 0.99 (0.16) | 1.03 (0.31) | 1.08 (0.35) | 0.570 (.455) | 1.631 (.210) | **0.96 (0.15)** | **0.99 (0.15)** | **1.14 (0.55)** | **1.48 (0.80)** | 1.202 (.281) | **4.135 (.050)** |
| Right & Down | 1.13 (0.24) | 0.96 (0.16) | 1.04 (0.17) | 1.02 (0.36) | 1.587 (.216) | 0.039 (.844) | **0.95 (0.08)** | **0.91 (0.07)** | **1.11 (0.22)** | **1.26 (0.66)** | 0.215 (.646) | **4.986 (.032)** |
| Down | 0.95 (0.15) | 1.04 (0.27) | 1.23 (0.54) | 1.17 (0.47) | 0.014 (.905) | 2.562 (.118) | 1.08 (0.24) | 0.98 (0.15) | 1.05 (0.13) | 1.17 (0.47) | 0.023 (.881) | 0.731 (.399) |
| Left & Down | 1.17 (0.57) | 1.24 (0.53) | 1.17 (0.51) | 1.10 (0.31) | 0.000 (.987) | 0.172 (.681) | 1.46 (0.46) | 1.04 (0.16) | 1.22 (0.56) | 1.15 (0.45) | 2.949 (.095) | 0.202 (.656) |
| Left | 1.03 (0.36) | 1.16 (0.44) | 1.13 (0.64) | 1.15 (0.43) | 0.259 (.614) | 0.097 (.757) | 1.31 (0.49) | 1.04 (0.20) | 1.16 (0.36) | 1.47 (0.85) | 0.024 (.877) | 0.610 (.441) |
| Left & Up | 1.02 (0.35) | 1.00 (0.25) | 1.11 (0.52) | 1.12 (0.48) | 0.002 (.963) | 0.645 (.427) | 1.38 (0.35) | 1.01 (0.23) | 1.20 (0.55) | 1.29 (0.69) | 0.649 (.427) | 0.097 (.758) |

**Table S6**. Normalised (to head only) left upper trapezius muscle activation levels of both sexes in four helmet conditions when pointing towards the nine environmental targets. Data are shown as mean (standard deviation) and statistical results are shown as F-value (p-value).

| **Left**  **UT** | Helmet | | | | | | Helmet plus NVG | | | | | |
| --- | --- | --- | --- | --- | --- | --- | --- | --- | --- | --- | --- | --- |
|  | No HBS | | HBS | | Main effect - Sex | Main effect - HBS | No HBS | | HBS | | Main effect - Sex | Main effect - HBS |
|  | M | F | M | F |  |  | M | F | M | F |  |  |
| Centre | 1.10 (0.65) | 0.98 (0.23) | 0.90 (0.30) | 1.28 (0.67) | 0.677 (.416) | 0.103 (.750) | 0.97 (0.33) | 1.11 (0.38) | 1.05 (0.45) | 1.01 (0.14) | 0.256 (.635) | 0.041 (.848) |
| Up | 1.41 (0.83) | 0.94 (0.16) | 1.24 (0.67) | 1.01 (0.36) | 3.750 (.061) | 0.098 (.756) | 1.11 (0.40) | 1.01 (0.34) | 1.20 (0.34) | 1.15 (0.48) | 0.322 (.575) | 0.805 (.376) |
| Right & Up | 1.03 (0.61) | 0.97 (0.29) | 0.99 (0.41) | 0.86 (0.20) | 0.534 (.470) | 0.339 (.564) | 1.45 (0.88) | 1.03 (0.33) | 0.91 (0.18) | 1.00 (0.17) | 0.883 (.355) | 2.757 (.107) |
| Right | 0.87 (0.54) | 0.88 (0.35) | 0.84 (0.34) | 1.15 (0.63) | 1.063 (.310) | 0.645 (.427) | 1.17 (0.44) | 1.27 (0.61) | 1.13 (0.61) | 1.29 (0.55) | 0.531 (.471) | 0.004 (.950) |
| Right & Down | 1.45 (0.99) | 0.95 (0.19) | 1.03 (0.25) | 0.94 (0.28) | 2.976 (.093) | 1.593 (.215) | 1.13 (0.24) | 1.51 (0.86) | 1.02 (0.27) | 1.15 (0.35) | 2.152 (.152) | 1.953 (.172) |
| Down | 1.04 (0.30) | 0.98 (0.20) | 1.05 (0.33) | 1.10 (0.36) | 0.000 (.994) | 0.518 (.477) | 1.04 (0.18) | 1.15 (0.48) | 1.11 (0.22) | 1.10 (0.30) | 0.229 (.636) | 0.010 (.921) |
| Left & Down | 1.07 (0.36) | 1.05 (0.36) | 0.96 (0.25) | 0.94 (0.17) | 0.030 (.864) | 1.384 (.247) | 1.07 (0.49) | 1.17 (0.75) | 0.91 (0.12) | 0.97 (0.20) | 0.296 (.590) | 1.440 (.239) |
| Left | 1.18 (0.48) | 1.03 (0.26) | 1.22 (0.48) | 0.97 (0.15) | 2.905 (.097) | 0.006 (.936) | **1.23 (0.58)** | **1.06 (0.24)** | **1.32 (0.41)** | **0.95 (0.20)** | **4.382 (.044)** | 0.010 (.922) |
| Left & Up | 1.12 (0.53) | 1.10 (0.37) | 1.08 (0.18) | 1.05 (0.18) | 0.046 (.831) | 0.130 (.720) | 0.99 (0.07) | 1.03 (0.22) | 1.29 (0.62) | 0.95 (0.08) | 1.611 (.214) | 0.786 (.382) |

**Table S7**. Normalised (to head only) right splenius capitis muscle activation levels of both sexes in four helmet conditions when pointing towards the nine environmental targets. Data are shown as mean (standard deviation. Statistical results are shown as F-value (p-value).

| **Right**  **SpC** | Helmet | | | | | | Helmet plus NVG | | | | | |
| --- | --- | --- | --- | --- | --- | --- | --- | --- | --- | --- | --- | --- |
|  | No HBS | | HBS | | Main effect - Sex | Main effect - HBS | No HBS | | HBS | | Main effect - Sex | Main effect - HBS |
|  | M | F | M | F |  |  | M | F | M | F |  |  |
| Centre | 0.95 (0.36) | 1.10 (0.43) | 0.88 (0.30) | 1.06 (0.27) | 2.286 (.139) | 0.276 (.603) | **1.09 (0.48)** | **1.60 (0.52)** | **1.01 (0.31)** | **1.34 (0.65)** | **7.379 (.035)** | **12.434 (.012)** |
| Up | 1.04 (0.30) | 1.22 (0.51) | 0.96 (0.24) | 0.91 (0.38) | 0.334 (.567) | 2.827 (.101) | 1.21 (0.36) | 1.27 (0.75) | 1.09 (0.48) | 1.28 (0.89) | 0.355 (.555) | 0.064 (.802) |
| Right & Up | 0.81 (0.29) | 0.87 (0.31) | 0.71 (0.28) | 0.67 (0.20) | 0.008 (.931) | 3.187 (.083) | 0.87 (0.28) | 0.97 (0.40) | 1.10 (0.51) | 1.15 (0.69) | 0.227 (.637) | 1.564 (.220) |
| Right | 0.68 (0.30) | 1.02 (0.39) | 0.66 (0.32) | 0.78 (0.58) | 2.937 (.096) | 0.943 (.338) | 1.06 (0.43) | 0.79 (0.17) | 0.88 (0.62) | 0.91 (0.40) | 0.664 (.421) | 0.038 (.847) |
| Right & Down | 1.16 (0.31) | 1.00 (0.36) | 0.92 (0.61) | 0.77 (0.35) | 1.358 (.252) | 2.884 (.098) | 1.06 (0.42) | 1.41 (0.85) | 1.01 (0.66) | 0.86 (0.15) | 0.276 (.603) | 2.364 (.134) |
| Down | 1.14 (0.25) | 1.21 (0.43) | 1.32 (0.78) | 0.85 (0.29) | 1.686 (.202) | 0.378 (.542) | 1.74 (0.63) | 1.51 (0.66) | 1.38 (1.02) | 1.40 (0.59) | 0.200 (.657) | 0.908 (.348) |
| Left & Down | 1.21 (0.51) | 1.09 (0.50) | 0.89 (0.31) | 0.93 (0.37) | 0.092 (.763) | 3.079 (.088) | 1.81 (0.59) | 1.53 (0.75) | 1.38 (0.66) | 1.16 (0.47) | 1.330 (.257) | 3.642 (.065) |
| Left | 1.06 (0.25) | 1.08 (0.45) | 0.95 (0.21) | 0.89 (0.31) | 0.042 (.839) | 1.959 (.171) | 1.97 (0.59) | 1.48 (0.78) | 1.47 (0.53) | 1.22 (0.45) | 3.398 (.075) | 3.602 (.062) |
| Left & Up | **1.09 (0.30)** | **1.13 (0.58)** | **0.94 (0.16)** | **0.80 (0.31)** | 0.209 (.650) | **4.267 (.046)** | 1.27 (0.21) | 1.39 (0.69) | 1.33 (0.40) | 1.10 (0.38) | 0.163 (.689) | 0.588 (.449) |

**Table S8**. Normalised (to head only) left splenius capitis muscle activation levels of both sexes in four helmet conditions when pointing towards the nine environmental targets. Data are shown as mean (standard deviation) and statistical results are shown as F-value (p-value).

| **Left**  **SpC** | Helmet | | | | | | Helmet plus NVG | | | | | |
| --- | --- | --- | --- | --- | --- | --- | --- | --- | --- | --- | --- | --- |
|  | No HBS | | HBS | | Main effect - Sex | Main effect - HBS | No HBS | | HBS | | Main effect - Sex | Main effect - HBS |
|  | M | F | M | F |  |  | M | F | M | F |  |  |
| Centre | 1.04 (0.34) | 1.08 (0.24) | 1.00 (0.31) | 0.98 (0.18) | 0.008 (.930) | 0.605 (.442) | **1.24 (0.46)** | **1.51 (0.31)** | **1.19 (0.43)** | **1.14 (0.39)** | 1.185 (.318) | **11.668 (.014)** |
| Up | 1.43 (0.88) | 1.16 (0.29) | 1.24 (0.77) | 1.25 (0.70) | 0.364 (.550) | 0.047 (.829) | 1.38 (0.57) | 1.31 (0.53) | 1.47 (0.91) | 1.50 (0.97) | 0.207 (.652) | 0.015 (.903) |
| Right & Up | 1.22 (0.59) | 1.20 (0.35) | 1.24 (0.47) | 0.90 (0.24) | 2.860 (.100) | 2.260 (.142) | 1.63 (0.69) | 1.72 (0.72) | 1.26 (0.62) | 1.33 (0.54) | 0.144 (.707) | 3.280 (.079) |
| Right | **0.97 (0.33)** | **1.60 (0.69)** | **1.05 (0.41)** | **1.16 (0.55)** | **5.112 (.030)** | 1.271 (.267) | **1.62 (0.42)** | **1.54 (0.53)** | **0.97 (0.17)** | **1.39 (0.76)** | 1.048 (.313) | **5.654 (.023)** |
| Right & Down | 1.56 (0.70) | 1.46 (1.02) | 1.32 (0.54) | 1.62 (1.13) | 0.134 (.717) | 0.022 (.884) | **1.28 (0.45)** | **1.73 (0.67)** | **0.91 (0.31)** | **1.03 (0.59)** | 2.635 (.115) | **9.238 (.005)** |
| Down | 1.77 (0.95) | 1.35 (0.40) | 1.14 (0.62) | 1.14 (0.85) | 0.518 (.476) | 3.537 (.068) | **1.25 (0.46)** | **2.03 (0.41)** | **1.09 (0.41)** | **1.50 (0.95)** | **8.066 (.008)** | 2.748 (.107) |
| Left & Down | **1.23 (0.61)** | **1.30 (0.79)** | **0.75 (0.29)** | **0.75 (0.30)** | 0.086 (.772) | **8.786 (.005)** | 1.36 (0.83) | 1.00 (0.45) | 1.04 (0.63) | 0.87 (0.29) | 1.729 (.198) | 1.182 (.285) |
| Left | 0.97 (0.41) | 1.27 (0.41) | 0.91 (0.58) | 1.14 (0.51) | 2.888 (.098) | 0.358 (.554) | 1.54 (0.91) | 1.61 (0.91) | 1.10 (0.31) | 1.41 (0.62) | 0.812 (.374) | 2.045 (.162) |
| Left & Up | 1.22 (0.94) | 0.98 (0.57) | 0.63 (0.32) | 0.86 (0.32) | 0.002 (.968) | 3.581 (.066) | 1.30 (0.45) | 1.31 (0.69) | 1.00 (0.59) | 1.28 (0.68) | 0.483 (.492) | 0.683 (.415) |
